# Loss of cilia causes embryonic lung hypoplasia, liver fibrosis, and cholestasis in the *talpid*^3^ ciliopathy mutant

**DOI:** 10.4161/org.28819

**Published:** 2014-04-17

**Authors:** Megan G Davey, Lynn McTeir, Andrew M Barrie, Lucy J Freem, Louise A Stephen

**Affiliations:** Division of Developmental Biology; The Roslin Institute and R(D)SVS; University of Edinburgh; Midlothian, UK

**Keywords:** TALPID3, KIAA0586, short-rib polydactyly, pulmonary hypoplasia, liver, cholestasis, cilia, ciliopathy, SHH, GLI

## Abstract

*Sonic hedgehog* plays an essential role in maintaining hepatoblasts in a proliferative non-differentiating state during embryogenesis. Transduction of the Hedgehog signaling pathway is dependent on the presence of functional primary cilia and hepatoblasts, therefore, must require primary cilia for normal function. In congenital syndromes in which cilia are absent or non-functional (ciliopathies) hepatorenal fibrocystic disease is common and primarily characterized by ductal plate malformations which underlie the formation of liver cysts, as well as less commonly, by hepatic fibrosis, although a role for abnormal Hedgehog signal transduction has not been implicated in these phenotypes. We have examined liver, lung and rib development in the *talpid^3^* chicken mutant, a ciliopathy model in which abnormal Hedgehog signaling is well characterized. We find that the *talpid^3^* phenotype closely models that of human short-rib polydactyly syndromes which are caused by the loss of cilia, and exhibit hypoplastic lungs and liver failure. Through an analysis of liver and lung development in the *talpid^3^* chicken, we propose that cilia in the liver are essential for the transduction of Hedgehog signaling during hepatic development. The *talpid^3^* chicken represents a useful resource in furthering our understanding of the pathology of ciliopathies beyond the treatment of thoracic insufficiency as well as generating insights into the role Hedgehog signaling in hepatic development.

## Introduction

Ciliopathies are multi-organ syndromes in which disorders arise either directly due to a loss of cilia formation, or from abnormal processes downstream of cilia function.^1^^,^^2^ Several organ systems which are affected in human ciliopathies such a Bardet-Biedl and Meckel syndrome, are associated with a loss of Hedgehog (Hh) pathway regulation during embryonic development, including polydactyly and abnormal bone formation.^3^ This is due to a requirement for primary cilia during Hh signal transduction when components of the Hh pathway such as the receptors PTCH1 and SMO are trafficked to and enriched in the cilia. ENU mutagenesis screens for Hh pathway components and loci mapping in human ciliopathy conditions have determined many proteins which are essential for cilia formation and function.^1^^,^^3^

Short-rib polydactyly (SRP) type III syndromes are a range of functionally, and genetically overlapping ciliopathies, presenting primarily with short ribs, reduced thoracic capacity, and pulmonary hypoplasia, leading to respiratory insufficiency and in severe cases, thoracic asphyxiation and death caused by constricted thoracic volume.^4^^-^^7^ Patients are also characterized by polydactyly, suggesting that Hh signaling is abnormal, as well as shortening of the long bones and metacarpels,^8^ cystic kidneys,^9^ liver fibrosis, and cholestasis.^10^ SRPIII syndromes are highly variable; although fetal lethality is common, children surviving infancy may undergo surgery to increase thoracic volume and live to adulthood, where they may present with combinations of key traits. In these cases, hepatic disease becomes more apparent, with liver transplants reported in patients as early as 7 years old.^11^

The Hh signaling pathway is well studied in the patterning and development of many organ systems. In the mouse lung *Shh* is necessary for early lung development^12^^,^^13^ and ablation of signaling with cyclopamine in the chicken, like the mouse, causes a loss of lung epithelial branching.^14^ Although Hh signaling has been shown to be abnormal in models of asphyxiating ciliopathies,^7^ a loss of lung morphogenesis has not been shown to be the primary cause of this.^15^ At the initiation of the developing liver bud, interactions between *Shh* and FGF signaling in the endoderm have been proposed to specify hepatic endothelial cells.^16^ Subsequently both *Shh* and *Indian hedgehog* ligands are expressed by hepatoblasts between E11.5-E17.5^17^ in the mouse, as well as the Hh responsive genes *Gli1*^17^ and *Ptch1*.^18^ Addition of Hh ligand in vitro causes an increase in hepatoblasts proliferation. Thus the current model for the action of Hh signaling during liver development is that it acts to control the balance between hepatoblast proliferation and the differentiation to hepatocytes.^17^ There is also a well-documented role for *SHH* in liver regeneration and repair; Hh responsive cells are observed in the adult liver when damaged.^19^^,^^20^ *SHH* is activated in response to chronic liver injury, but in addition, increasing Hh signaling through reduction of PTCH1 activity, results in greater damage to the liver.^20^ Conversely, inhibition of the Hh pathway has also been shown to reverse the development of fibrosis and hepatocarcinoma.^21^ Hh signaling has therefore become a focal point for understanding liver repair, regeneration and the basis of various liver cancers.^21^^-^^24^ The role of cilia in transduction of the Hh signal within the liver has not been investigated, although ciliated cells correspond to the intrahepatic Hh responsive cells in adult mice.^20^ We can therefore assume, as in all other cells types investigated, that Hh responsive liver cells require cilia to transduce the Hh signal. Cilia have other functions within the developing liver; a loss of cilia on cholangiocytes, which localize proteins such as the polycystin family, important in mechano-, osmo-, and chemo-sensory functions, leads to cystic and fibrotic liver diseases.^25^^,^^26^ The severity of most ciliopathy models commonly results in embryonic lethality; however, the role of cilia in developmental hepatic phenotypes is particularly under-studied.

The *talpid^3^* chicken provides a classic model for studying human ciliopathies and Hh signaling, exhibiting many ciliopathy phenotypes, including polydactyly,^27^ polycystic kidneys^28^ and a loss of endochondral bone ossification^29^ and has been useful in elucidating the role of Hh signaling in limb and neural tube development.^27^^,^^30^ The TALPID3 protein (KIAA0586) localizes to the centrosome in human, chicken, mouse and zebrafish and is required for the docking of the basal body prior to ciliogenesis.^28^^,^^31^^,^^32^ Loss of TALPID3 protein causes a loss both of motile and non-motile primary cilia.^28^^,^^33^ Due to the loss of primary cilia in TALPID3^−/−^ cells, the downstream effectors of Hh signaling, the GLI transcription factors are abnormally processed and localized and their function therefore abrogated. Consequently, as is seen commonly in other ciliopathy models, the expression of *PTCH1* is not initiated at sites of high Hh signaling.^27^^,^^31^

Here we propose that the classical *talpid*^3^ chicken may act as a model for the SRPIII class of ciliopathies. The *talpid^3^* chicken is able to develop until E7-E12, substantially further than the *Talpid3^−/−^* mouse and most other mammalian ciliopathy models, allowing us to extend our analyses to organs not possible in the mouse, and thus to study the role of Hedgehog signaling in the developing lung and liver.

## Results

### *Talpid^3^* embryos exhibit abnormal liver and lung morphology reminiscent of SRPIII patients

Gross morphological analysis of *talpid^3^* embryos identified clear abnormalities in the liver and lungs (Fig. 1). Birds differ from mammalian lung development, in that they have a parabronchial lung, rather than the alveolar lung found in mammals. However branching events in avians are similar to the mammalian lung and also exhibit conserved signaling pathways.^34^ By E8 the *wt* lungs are highly branched, distinct structures (Fig. 1A), whereas *talpid^3^* lungs are smaller, lacking branches, and were typically surrounded by fibrotic mesenchymal tissue (Fig. 1B). The abdominal air sac is however normal (Fig. 1A and B, asterisk).

**Figure:**
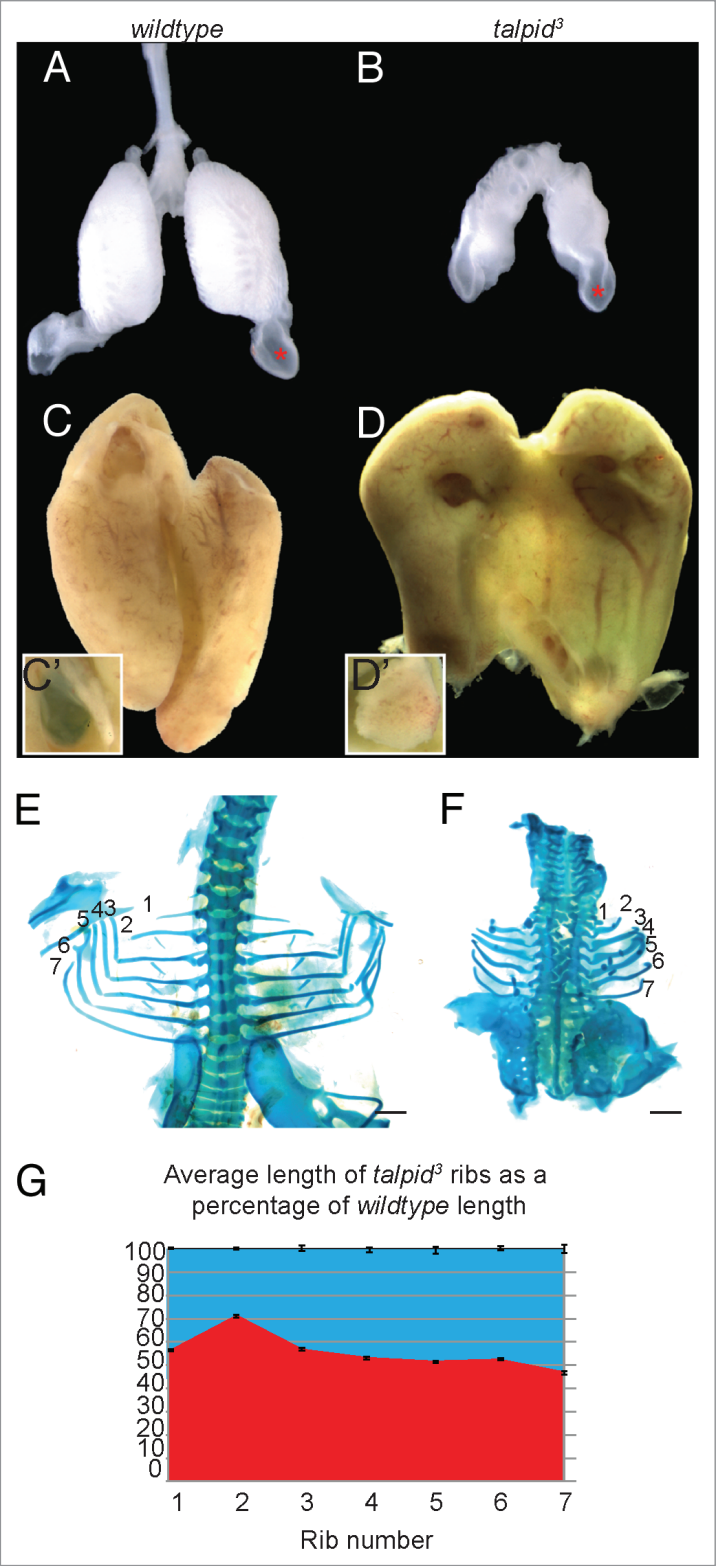
**Figure 1.** The *talpid^3^* chicken exhibits abnormal lung and liver morphology. Compared with E10 *wt* lung (**A**), the *talpid^3^* lung is smaller and poorly branched (**B**), air sac development was normal (red asterisk) (**A andB**). *Wt* liver (**C**) and *talpid*^3^ liver (**D**) are of similar size, although the *talpid^3^* liver is green. The *wt* gall bladder is bile filled (green) (**C’**), while the *talpid^3^* gall bladder lacks bile (**D’**). At E10 individual ribs were measured in *wt* (**E**) and compared with the corresponding rib in *talpid^3^* (**F**). Average lengths were reduced in the *talpid^3^* chicken; Rib one = 44% reduced, rib two = 28%, rib three = 44%, rib four = 46%, ribs five/six = 48%, rib seven = 53% smaller in *talpid^3^* (**G**). Magnification is the same between **A and B**, **C and D**, and **E and F**.

All *talpid^3^* embryos dissected had ventral abdominal herniation of viscera. All *talpid^3^* livers were normally patterned with a lobe either side of the midline, the right lobe being larger and the left exhibiting a fissure dividing the right lobe into two parts. However, the *talpid^3^* liver was clearly distinguished from the *wt* (Fig. 1C) by its green color (Fig. 1D), suggesting increased levels of bile within the *talpid^3^* liver. The gall bladder, while visible in *talpid^3^* and correctly attached to the right liver lobe, did not contain bile (compare Fig. 1C' with Fig. 1D'). SRPIII conditions in humans are associated with abnormal thoracic skeleton development, in particular, short ribs. The *talpid^3^* rib cage was considerably smaller than that of the *wt* (Fig. 1E and F) and individual ribs measured between 44–53% of the length of their *wt* counterparts (Fig. 1G).

### Lung development in *talpid^3^* chicken is abnormal

Pulmonary insufficiency is a key feature of SRPIII syndromes, but it is unclear if this is due to a primary effect on lung development or secondary to thoracic restriction. Having identified pulmonary hypoplasia in *talpid^3^* dissections, we characterized the extent of abnormalities in *talpid^3^* pulmonary development by histological analysis (Fig. 2). At E7, the primary *wt* mesobronchi consist of a thickened epithelium (Ep, Fig. 2B) surrounded by condensed subepithelial mesenchyme (SeM, Fig. 2B). The submesothelial mesenchyme comprising the remaining lung (SM, Fig. 2B) is punctured extensively by epithelial bronchiolar branches (asterisks Fig. 2A). Like SRPIII patients, *talpid^3^* lung development was highly variable; from embryos exhibiting two lungs that resemble small lungs with some mesobronchial branching (Fig. 2D), ranging to absent or extremely reduced lungs with no mesobronchi (not shown). A separated esophagus and trachea were observed in all samples. In less severely affected E7 *talpid^3^* lungs in which epithelia-lined lumen were present (Fig. 2D), bronchiolar lumen were smaller and unevenly distributed through the mesenchyme (arrows, Fig. 2D), the bronchiolar epithelia was thin and disorganized (Ep, Fig. 2E), and SeM condensations were not observed (Fig. 2D and E). Treatment of chick lungs at E8 with SHH pathway inhibitor cyclopamine causes a similar hypoplastic lung phenotype.^14^ We examined *SHH* expression in E6 chicken lungs (Fig. S1) and detected *SHH* in the *wt* trachea (Fig. S1A and B, arrow) but not in the mesobronchial epithelium, instead observing *SHH* expression in the *wt* distal lung mesenchyme (Fig. S1C, arrow). Neither the *talpid^3^* lung epithelium nor mesenchyme expressed *SHH* at E6 (Fig. S1D–F). *WNT5A* negatively regulates *SHH* expression in the chick lung and overexpression of *WNT5A* causes pulmonary hypoplasia.^14^ At E8 we observed *WNT5A* expression in both the distal bronchial epithelia and mesenchyme, whereas expression appeared reduced in the distal mesenchyme of *talpid^3^* lungs. *GATA6* is strongly expressed in the distal lung epithelia in humans^35^ and abnormal expression has been suggested as a cause of respiratory distress in neonates. At E8 *GATA6* was strongly expressed in distal epithelium of *wt* and *talpid^3^* embryos, confirming the distal epithelial identity of the *talpid^3^* bronchioles (Fig. S1G–J). We have previously shown that *FOXJ1*, a master regulator of motile ciliogenesis, is not expressed in the chicken embryo respiratory tract before E10, suggesting the developing lung epithelia does not have motile cilia at this time.^33^ We examined the presence and type of cilia at E10; short, primary cilia were identified in the three main tissue types of the *wt* lung; SeM, SM and epithelial (Fig. 2C). No cilia were identified in *talpid^3^* lungs (Fig. 2F). In summary due to a loss of cilia and cilia transduced pathways such as SHH, lung morphogenesis was severely disrupted, resulting in defects in both the epithelium and mesenchyme, although distal structures were still present.

**Figure:**
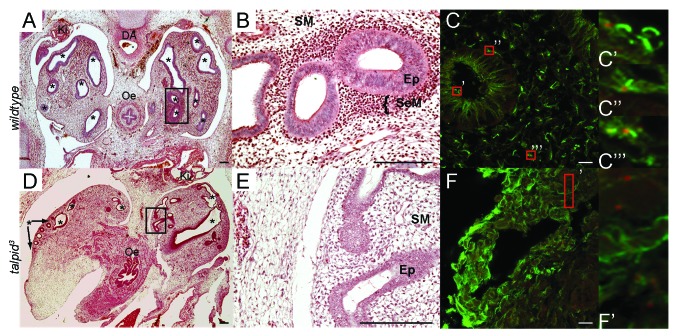
**Figure 2.** Lung development in the *wt* and *talpid^3^* chicken. Haematoxylin and eosin staining E7 (**A–E**). IHC anti-acetylated tubulin (cilia axonemes;green) and anti- γtubulin (centrosomes;red) E10 (**CandF**). At E7 the epithelial mesobronchi of each lung has branched extensively throughout the *wt* lung mesenchyme (asterisks) (**A**). The more mature *wt* primary bronchi (circled area) (**A andB**) are surrounded by a condensation of SeM cells (**B**). Mesobronchi branching in the *talpid^3^* lung is disturbed and differs between lungs within the same embryo (bronchi labeled with asterisk or arrow asterisk) (**D**), the epithelia is thinner and disorganized and no SeM condensations are seen (**E**). E10 *wt* lung exhibit cilia (**C**) on epithelia (**C’**), SeM (**C”**) and SM cells (**C”’**). E10 *talpid^3^* lung, cilia do not project from centrosomes (red box) (**F and F’**) Magnification comparable between **A–E**, **C–F**.

### The *talpid^3^* liver exhibits abnormal biliary tract development

*Wt* avian livers at E10 are highly organized structures, consisting of compact systems of hepatocytes surrounding blood vessels and sinusoidal spaces in which an immature version of the classic hepatic triumvirate can be observed, with cholangiocytes arranged in a ring, producing early bile ducts (Fig. 3A and B, asterisk C). In comparison, blood vessels are less clearly defined in the *talpid^3^* liver and sinusoidal spaces are considerably smaller, giving the liver a more condensed appearance overall (Fig. 3D). The ductal plate in the *talpid^3^* liver is considerably more cell dense with several layers of cholangiocytes forming along the ductal plate (Fig. 3E and F). Immunohistochemistry for cytokeratin19, expressed in the ductal plate (bile ducts) at E10 shows an increase in the number of epithelial cells in the *talpid^3^* ductal plate, (Fig. 3, red arrow F’ compared with C’) characteristic of a ductal plate malformation (DPM). This may well be caused by hyperplasia or abnormal remodelling and morphogenesis of the *talpid^3^* ductal plate. Bile in the *wt* liver sits within the lumen of the biliary duct (Fig. 3C, arrow), while bile is found between cholangiocytes and outside of the bile duct in *talpid*^3^ (Fig. 3F, arrow). At E12 fibrosis can be observed in *talpid^3^* livers around the ductal plate (Fig. 3L, blue; compare with *wt*Fig. 3I) and necrosis is widespread (n; Fig. 3J and K).

**Figure:**
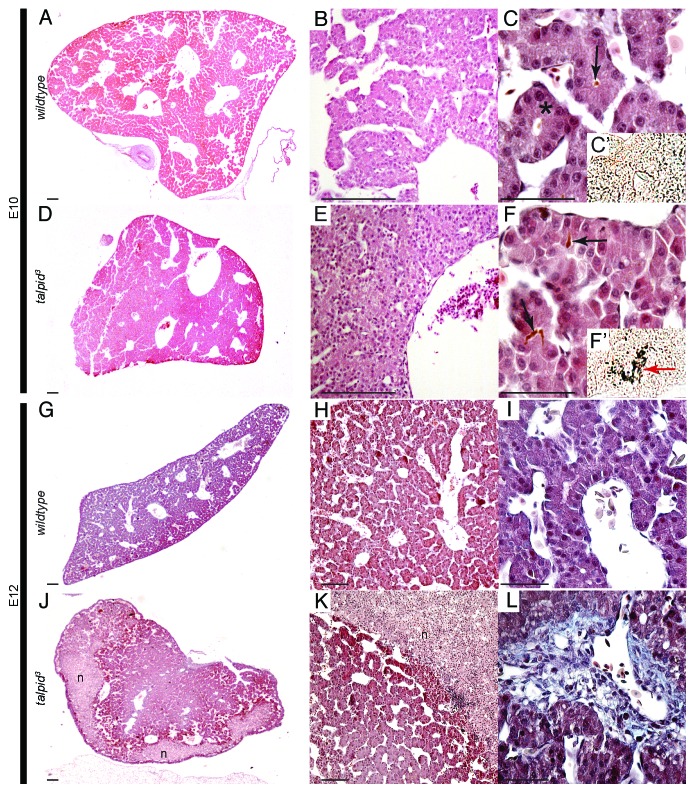
**Figure 3.** Abnormal liver histology in the *talpid^3^*chicken. Haematoxylin and eosin staining E10 (**A–F**), E12 (**G**, **H**, **J**, **K**). IHC Cytokeratin19 (**C’ andF’**). Masson’s trichrome staining at E12 (**IandL**). E10, *wt* livers have large spaces throughout (**AandB**) and exhibit an immature version of the classic hepatic triumvirate with cholangiocytes arranged in a ring to produce early bile ducts (asterisk) (**C**) with bile (arrow) (**C**) but no Cytokeratin19 present (**C’**). E10 *talpid^3^* liver is more compact, with fewer, smaller, spaces (**D andE**). Bile ducts develop but are overcrowded with cholangiocytes (**F**) which express Cytokeratin19 (**F’**) and bile is observed between the cholangiocytes (arrow) (**F**). E12 *wt* liver is more compact (**G andH**). E12 *talpid^3^* liver presents areas of necrosis (**J**, **K**) and portal fibrosis (blue stain) (**L**) compared with *wt* (**I**).

### *PTCH1* is expressed in the normal embryonic liver

Long cilia have been observed on cholangiocytes during human liver development and a loss causes liver defects in fetuses with Meckel syndrome, a severe ciliopathy.^36^ To observe if cilia are only found on cholangiocytes during development we used immunohistochemistry to observe cilia in the developing liver. Short primary cilia were widely observed in the *wt* liver as early as E6 (Fig. 4A, circled), resembling the short primary cilia which are required for Hh signaling elsewhere in the embryo, rather than long cilia that have been reported on cholangiocytes.^23^ In *talpid^3^* liver tissue, although centrosomes were observed (Fig. 4B, circled), cilia were absent. Loss of cilia in the liver suggests that hepatic abnormalities in the *talpid^3^* liver may be due to aberrant SHH signaling. Levels of *PTCH1* expression indicate levels of Hh signaling, therefore Real-time qPCR was used to study levels of *PTCH1* as a read out for SHH activity in the liver at E6, prior to onset of fibrosis and necrosis. *PTCH1* expression was reduced 0.08-fold in the *talpid^3^* liver compared with *wt* (Fig. 4C), suggesting Hh signaling is greatly abrogated in the *talpid^3^* embryonic liver.

**Figure:**
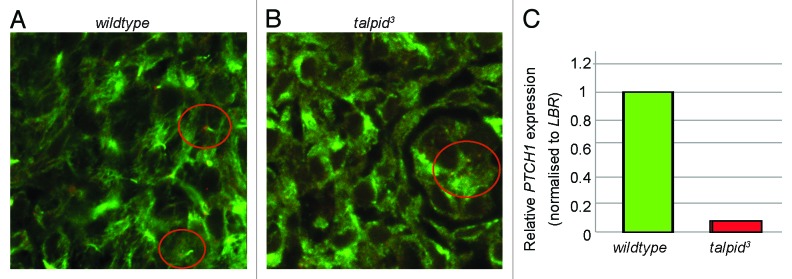
**Figure 4.** Hedgehog signaling is perturbed in the *talpid^3^* liver during development. IHC anti-acetylated (cilia axonemes, green) and γtubulin (centrosomes, red) E6 (**A andB**). *Wt* liver has cilia axonemes projecting from centrosomes (circled) (**A**) cilia axonemes are not observed projecting from centrosomes in the *talpid*^3^ liver (circled) (**B**). (**C**) Real-time PCR identified a 0.08-fold reduction in *PTCH1* expression in the day 6 *talpid^3^* liver, compared with the *wt*.

## Discussion

### The *talpid^3^* chicken models human SRPIII syndromes

Abnormal development in the *talpid^3^* chicken offers an insight into human development and disease. SRPIII syndromes encompass a variable spectrum of developmental disorders, many of which are related to ciliopathies. We suggest that the *talpid^3^* chicken phenocopies a number of these, including short ribs, polydactyly, polycystic kidneys, liver fibrosis and cholestasis, making it a useful model to study SRPIII and related syndromes.

### Does chicken lung morphogenesis model mammalian lung morphogenesis?

With some exceptions^14^^,^^34^^,^^37^^,^^38^ the majority of our understanding of the signaling driving pulmonary development has been investigated by studies in mammalian systems.^39^^,^^40^^,^^65^ However, the chicken is a useful alternative to mammalian models, offering shorter gestation times and many alternative options for embryonic manipulations (for review see ref. 41). If we are to fully utilize the chicken as a system for understanding human development and disease, it is important to determine the similarities and differences between them. *Shh* signaling is essential for early mouse pulmonary development^12^^,^^13^ where it is expressed in the distal epithelial endoderm at the budding tips.^42^ Likewise, the Shh receptor, *PTCH1* is expressed in the distal mesenchyme, in both mouse^43^ and human^44^ suggesting that a requirement for *SHH* signaling in the growth and differentiation of the bronchioles is conserved within mammalian species. Perturbation of the *SHH* signaling pathway through mutations in *SHH* and the downstream targets, *GLI2* and *GLI3,* results in highly abnormal tracheal and pulmonary development, in particular loss of asymmetry and pulmonary hypoplasia.^45^^,^^46^ In contrast although we found strong *SHH* expression in the avian gut, as described previously,^47^ expression of *SHH* in the budding epithelium of the lungs was absent from wild type chickens, although we detected *SHH* expression in distal mesenchyme. This suggests that while the target tissue of SHH action is the same between mouse and chicken (the distal mesenchyme), the chicken alters its expression between an early mesenchymal domain to a later epithelial expression,^14^ during development. While there are clear differences in the basic development of avian and mammalian lungs, analysis of epithelial and mesenchymal expressed genes at later stages of lung development in the *wt* and *talpid^3^* chicken also suggest that many of the underlying processes are still comparable to the mammalian model. Previous studies in mouse have shown *Nkx2.1, Gata6, Sox2, Wnt5a,* and *Wnt3a* to be expressed in the branching epithelia. We have confirmed this in the avian model and further identified that this expression was not significantly disrupted in the *talpid^3^* mutant. It is however clear that branching is affected in the *talpid^3^* chicken, albeit to a variable extent. Most interestingly, we identified a reduction of mesenchymal *WNT5a* in *talpid^3^* embryos. In the mouse and chicken, *Wnt5a* is thought to regulate *Shh* and *Fgf10* in the developing lung^14^^,^^48^ and in turn *Fgf10* null mice fail to produce any structures distal to the primary bronchi.^49^ Loss of *WNT5A* would be expected to result in an increase in SHH signaling.^14^ However, in the *talpid^3^* chicken, the loss of WNT5A accompanies a loss of SHH phenotype, hypoplastic lungs. We suggest that this is due to a complex signaling loop that we are only beginning to understand. The increase in SHH caused by loss of WNT5A, previously reported by Loscertales and colleagues,^14^ is caused by an increase in GLIA and loss of GLIR, producing a hyperplastic lung phenotype. Loss of SHH signaling in the same report, was achieved by cyclopamine, a Hedgehog repressor that inhibits the GLIA pathway, resulting in hypoplasia. The *talpid^3^* chicken phenotype is the result of a loss of GLIA and GLIR,^27^ producing a hypoplasia phenotype. We propose that a WNT5A/SHH signaling loop acts within a PCP/cilia network to maintain development in the lung. The loss of cilia in the *talpid^3^* mutant has previously been attributed to abnormal basal body migration,^33^ a PCP phenotype. It is likely that this lack of cilia not only prevents the tissue from responding to loss of SHH signaling, but also to loss of WNT5A, therefore producing a loss of SHH phenotype, despite the apparent loss of WNT5A.

Many mouse mutants with a loss of *SHH* signaling exhibit a tracheoesophageal fistula, whereby the early esophagus fails to split to produce discrete tracheal and esophageal tubes.^12^ In contrast, in the *talpid^3^* embryo, the trachea was clearly distinct from the esophagus in all animals studied. Either this may be because *SHH* is not required for avian respiratory tract development, or alternatively may be due to loss of *GLI* processing observed in *talpid*^3^, which in some organs causes loss of Hh phenotype, and in others a gain of Hh phenotype.^50^ Hh signaling requires interaction with GLI proteins in both activator (A) and repressor (R) form; therefore loss of GLIR in the *talpid*^3^ limb results in polydactyly, (due to loss of repression of Hh signaling targets by GLIR), while craniofacial abnormalities can be attributed to a loss of GLIA but are partially rescued by lack of GLIR also, producing variable holoprosencephaly that is less severe than *SHH*^−/−^ mutants.^27^^,^^51^

### Implications for SRPIII patients

The analysis of the molecular basis of lung hypoplasia, a disruption identified in infants with SRPIII syndromes, is unique to this study and has rarely been studied in models for the disease.^4^ Analysis of SRPIII mouse models caused by mutations in *Ift80* and *Ift144* (in which lung development is not assessed) indicate, as in *talpid^3^*, that aberrant Hh signaling is the cause of many SRPIII associated phenotypes, such as polydactyly.^5^^,^^15^ A further model of SRPIII, the *Wdr35* mouse does demonstrate pulmonary hypoplasia which is independent of rib development.^52^ We therefore suggest that lung hypoplasia identified in the *Wdr35* mutant, *talpid^3^* and SRPIII patients may be due to Hh signaling abnormalities and is not solely a secondary consequence of physical constriction due to thoracic dystrophy as is generally assumed in SRPIII patients. Treatment of SRPIII patients often involves expansion of the rib cage to allow pulmonary growth, while this treatment offers a great increase in life expectancy, we know of no studies that have investigated how well pulmonary development is rescued, and we would suggest that surgical intervention will never fully restore pulmonary function in these patients due to inability of the lungs to undergo normal morphogenesis due to Hh signaling defects.

Among the key difficulties facing patients who survive to adulthood is the development of fibrocystic kidneys,^53^^-^^55^ which has previously been described in the *talpid^3^* chicken. Poor liver function has been recorded as early as 3 days of life in patients with the SRPIII syndrome Jeune’s Asphyxiating Dystrophy (JAD) with biopsies indicating portal fibrosis and dilated bile ducts.^11^ While the key presentation of JAD is asphyxiating thoracic dystrophy, as treatment improves it is important to recognize the range of hepatic abnormalities patients are susceptible to. Alongside fibrosis and DPMs described here, previous clinical reports have identified hepatomegaly^9^^,^^56^ and biliary cirrhosis.^11^ Fibrocystic liver presentations in Bardet-Biedl syndrome patients are reviewed in depth by Waters et al.,^57^ while the COACH subset of Joubert syndrome patients exhibit a very mild liver phenotype with portal hypertension, congenital fibrosis and mild DPM (reviewed ref. 58). In this study we have identified that ductal plate malformation (DPM) and cholestasis precede embryonic portal fibrosis in *talpid^3^* embryos. Extrahepatic biliary atresia, most likely caused by abnormal morphogenesis of the extrahepatic bile duct,^59^ has also been discussed in animal models for situs inversus and abnormal SHH signaling and may be a potential cause of bile retention, and subsequently hepatic necrosis.^60^^-^^63^ We propose however, that reduction in biliary duct lumen, caused by over-proliferation or abnormal remodelling of the ductal plate may be a cause of biliary blockage, while the loss of mechanosensory cholangiocyte cilia may prevent regulation of biliary flow, resulting in cholestasis.^25^^,^^64^ Cholestasis itself may therefore prove useful as either in identification of disease prior to liver damage in patients or as a target for therapy in reducing liver damage.

### The *talpid^3^* chicken indicates a requirement for Hh signaling via cilia in liver development

We have shown that short cilia are present and *PTCH1* is normally expressed in the developing liver indicating that Hh signaling is active in the embryonic liver and likely to be important in morphogenesis. We would predict a loss of *PTCH1* in *talpid^3^* livers, as we see in other *talpid^3^* embryonic tissues, due to a loss of cilia and Hh signal transduction.^27^^,^^56^ In *talpid^3^* embryos we have observed cholestasis, DPM and liver fibrosis. Although liver fibrosis has previously been linked to over-activation of the Hh pathway,^20^ it is not clear if these phenotypes are caused only through misregulation of Hh signaling in *talpid^3^* or more generally due to a loss of cilia. Cilia play numerous roles in liver homeostasis, including mediating functions of the cholangiocytes through the polycystins and other signaling pathways^65^ but certainly Hh signal transduction is defective in *talpid^3^* and this may prove a useful model for examining the function of Hh signaling in liver development and function.

## Concluding Remarks

A loss of cilia during embryonic development causes cholestasis and liver fibrosis. The *talpid^3^* chicken offers a valuable resource in understanding the role of Hh signaling in liver development and in furthering studies on SRPIII ciliopathies.

## Materials and Methods

### Embryo incubation, dissection and histology

Eggs from *talpid^3^* flock (MG Davey; *talpid^3^* chicken lines are maintained at the Roslin Institute under UK Home Office license 60/4506 [Dr Paul Hocking], after ethical review) were incubated at 38 °C for 6–12 d, staged as per ref. 66. Embryos were dissected into PBS, fixed 4%PFA.

### Histology

Fixed lung and liver samples were embedded in paraffin, and sectioned stained in hematoxylin and eosin, as per ref. 28 and Masson’s trichrome.

### Immunohistochemistry

Embryos were dissected into PBS, fixed, and organs of interest removed before sectioning as per reference 27. Immunohistochemistry was then performed as per reference 27. Antibodies used- acetylated α tubulin (Sigma-Aldrich T7451), γtubulin (Sigma-Aldrich T5192), anti-cytokeratin19 (Developmental Studies Hybridoma Bank Troma-III) anti-mouse (Life Technologies A11017), anti-rabbit (Life Technologies A21207).

### Alcian green staining

E10 embryos were dissected in ice-cold PBS, decapitated and eviscerated, and fixed overnight in 5% trichloroacetic acid. Embryos were then transferred into 0.1% alcian green/70% ethanol/1% HCl for 24hrs. Post dehydration, tissue was cleared using methyl salicylate. The rib cage was dissected, photographed and rib measurements taken using Image J. Rib one is often missing from the *talpid^3^* chicken, or too small to be measured.

### Whole-mount in situ hybridization

RNA probe synthesis and whole-mount RNA in situ hybridization was performed on lungs as per references 67 and 68. Photography was performed using a Leica M28 microscope. Chicken ESTs were obtained from previously used cDNA sequences or the BBSRC ChickEST Database^69^ collection held by ARK Genomics corresponding to- *GATA6* (ChEST944e13), *NKX2.1* (ChEST763 g11), *SOX2* (ChEST878b12), *WNT3A* (ChEST36j7), and *WNT5A* (ChEST378m15), *SHH*.^68^

### Real-time PCR

RNA was isolated from E6 livers using tri reagent (Sigma) and reverse transcription performed using High Capacity cDNA Reverse Transcription Kit (Applied Biosystems). Real-time PCR was performed using Brilliant III ultrafast SYBR green QPCR master mix (Agilent Technologies) and analysis performed using the stratagene MX3000 and MxPro software.

## Supplementary Material

Additional material

## Abbreviations:

PTCH1: patched 1
SMO: smoothened
Hh: hedgehog
SRPIII: short-rib polydactyly type III syndrome
SHH: sonic hedgehog
ENU: N-ethyl-N-nitrosourea
FGF: fibroblast growth factor
ChEST: chicken expressed sequence tag
wt: wildtype
Oe: oesophagus
Ki: kidney
DA: dorsal aorta
Ep: epithelium
SeM: subepithelial mesenchyme
SM: submesothelial mesenchyme
AS: air sac
IHC: immunohistochemistry
DPM: ductal plate malformation
Ift80/144: intraflagella transport protein 80/144
JAD: Jeune Asphyxiating Dystrophy
COACH: cerebellar vermis hypoplasia, oligophrenia, ataxia, colobomas, and hepatic fibrosis

## Notes

10.4161/org.28819
