# Supplementary material for: Loss of cilia causes embryonic lung hypoplasia, liver fibrosis, and cholestasis in the talpid3 ciliopathy mutant
Source: Organogenesis. 2014 Apr 17;10(2):177–85. doi: 10.4161/org.28819 (PMC4154951; doi:10.4161/org.28819)
Supplement: Additional material [file org-10-177-s01.pdf]

Landes Bioscience www.landesbioscience.com

Supplemental Material to:

Megan G Davey, Lynn McTeir, Andrew M Barrie,
Lucy J Freem, and Louise A Stephen

Loss of cilia causes embryonic lung hypoplasia, liver fibrosis and cholestasis in the *talpid*^{3} ciliopathy mutant

Organogenesis 2014; 10(2)
http://dx.doi.org/10.4161/org.28819

http://www.landesbioscience.com/journals/organogenesis/article/28819/

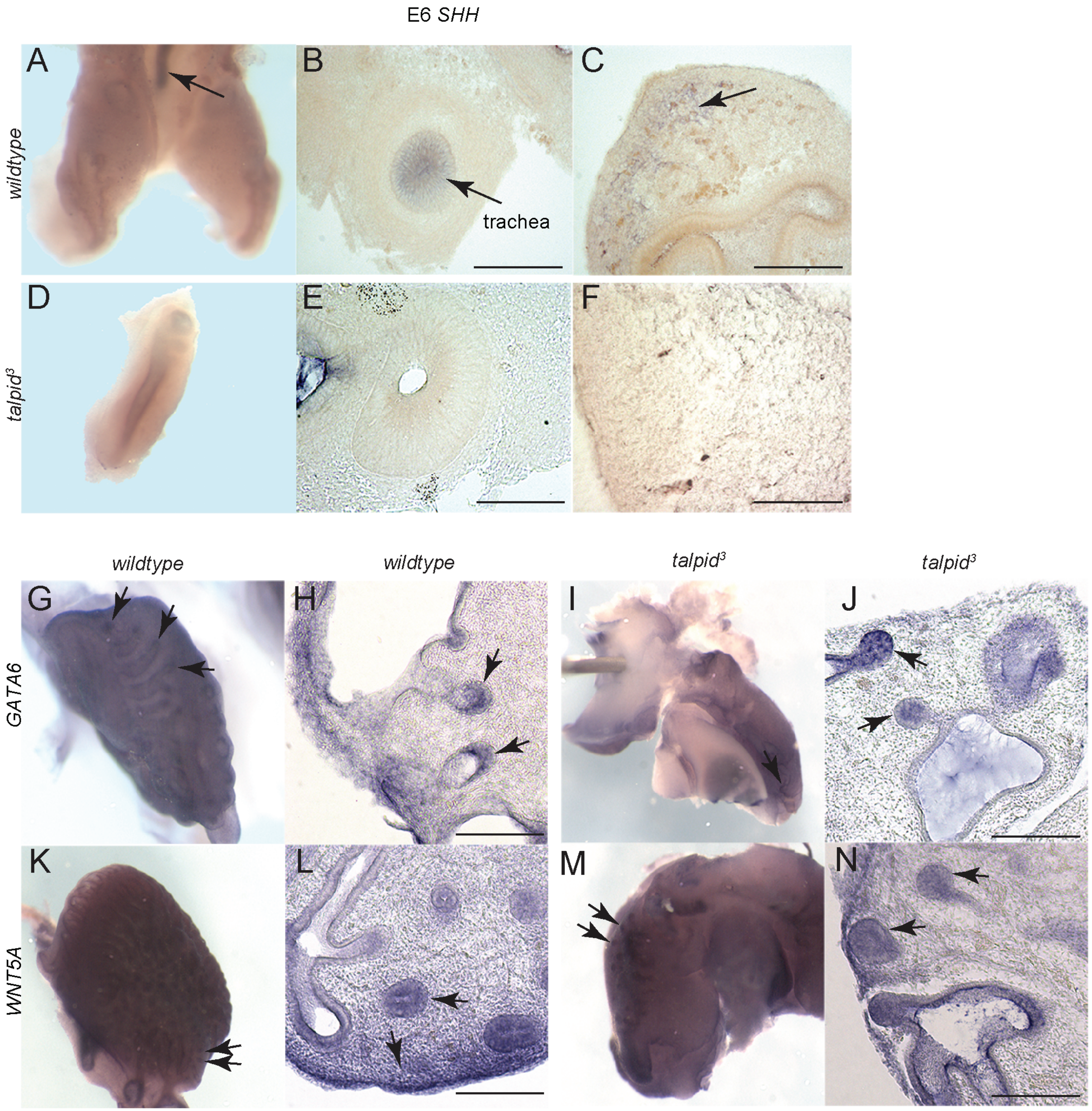

Supplemental Figure 1:
***SHH* expression is lost in *talpid³* lung development but distal epithelial markers are present**

Figure S1. SHH expression is lost in *talpid*^{3} lung development but distal epithelial markers are present. RNA in situ hybridisation for SHH (**A-F**), GATA6 (**G-J**), WNT5A (**K-N**). SHH expression in the wt trachea (arrow, **A** and **B**) and distal lung mesenchyme (**C**). SHH expression is not observed in *talpid*^{3} wholemount lung (purple stain is non-specific trapping in epithelial lumen, **D**), lung epithelium (**E**), or distal mesenchyme (**F**). GATA6 expression in wt wholemount lung, arrows indicating epithelium (**G**), in section expression is seen in distal mesenchyme and distal epithelial buds (arrows, **H**). GATA6 expression in *talpid*^{3} wholemount lung (**I**), in section, arrows indicating expression in distal buds, expression is not seen in mesenchyme (**J**). WNT5A expression in wt wholemount lung, arrows indicating epithelium (**K**), in section expression is seen in distal mesenchyme and distal epithelial buds (arrows, **L**). WNT5A expression in *talpid*^{3} wholemount lung (arrows, **M**), in section, arrows indicating expression in distal buds, expression is reduced in mesenchyme (**N**).
